# Universal ocular screening of 481 infants using wide-field digital imaging system

**DOI:** 10.1186/s12886-018-0943-7

**Published:** 2018-10-30

**Authors:** Yan Ma, Guangda Deng, Jing Ma, Jinghua Liu, Songfeng Li, Hai Lu

**Affiliations:** Department of Ophthalmology, Beijing Tongren Eye Center, Beijing Tongren Hospital, Capital Medical University, Beijing Ophthalmology and Visual Sciences Key Lab, 1 Dongjiaominxiang, Dongcheng District, Beijing, 100730 China

**Keywords:** Infant, Ocular screening, Wide-field imaging, RetCam

## Abstract

**Background:**

Universal ocular screening of infants is not a standard procedure in children’s health care system in China. This pilot study investigated prevalence of ocular abnormalities of 6 weeks-age infants using wide-field digital imaging system.

**Methods:**

Infants aged 6 weeks around were consecutively enrolled in a public hospital between April 2015 and August 2016. All the infants who were enrolled in the study underwent vision assessment, eye position examination, external eye check, pupillary light reflex, red reflex examination, anterior and posterior ocular segments were examined using flashlight, ophthalmoscope, and wide-field digital imaging system.

**Results:**

A total of 481 infants at 45.1 ± 6.1 days after birth were enrolled in the study. 198 infants had abnormal findings (41.2%). Retinal white spots and retinal white areas were the most common findings (42.9% of abnormalities and 17.7% of all infants screened). The second major finding was retinal hemorrhage (16.2% of abnormalities and 6.7% of all infants screened). Other abnormal findings include retinal pigmentation, concomitant exotropia, neonatal dacryocystitis, retinopathy of prematurity, ‘albinism-like fundus’, congenital nasolacrimal duct obstruction, familial exudative vitreoretinopathy, immature retina, corneal dermoid tumor, large physiologic cupping of optic disc, congenital persistent pupillary membrane, entropion trichiasis, subconjunctival hemorrhage, congenital cataract, vitreous hemorrhage, ptosis and choroidal nevus. Intervention of any form was required in 22 infants, which accounted for 11.1% of abnormalities detected and 4.6% of all infants screened.

**Conclusion:**

Universal ocular screening is not only necessary for preterm infants but also for full-term infants. Addition of red reflex examination with wide-field digital imaging system can enhance the sensitivity of screening for ocular fundus abnormities. Further study with a long-term follow-up is needed in the future.

## Background

Ocular problems in infants, especially visual impairment has to be detected passively since infants cannot convey their discomfort verbally. The American Academy of Pediatrics recommends red reflex examination as a component of ophthalmic evaluation of children. Ideally it should be started in the neonatal period and continued during the routine periodic well health visits by the children’s primary care provider. [1]. During the routine well health visits if any abnormality is detected the primary care provider should refer the child to an ophthalmologist. Currently in China and other developing counties, ocular screening for infants is not a strict requirement during the well health check-ups.

In China, infants go through their first well child check-up at about six weeks of age in some big cities and tertiary hospitals. But even in these places, sometimes the pediatricians are not comfortable using the ophthalmoscope to perform red reflex examination in infants and thus in most cases only an external examination of the eye is performed during the well child check-up. Therefore, some full-term infants with eye diseases are not detected in a timely manner. The condition can worsen and result in impaired vision. In some instances, by the time parents become aware of the visual impairment in their children, the ideal time for treatment would have been missed and it could result in life-threatening consequences.

In recent years, as children’s healthcare system in China has improved, policies about eye screening and vision care in childhood has been issued. Children in some metropolitan cities have received neonatal eye screening using wide-field digital imaging [2]. Most of these screenings were done in the first 72 h after birth. Previous studies showed that majority of abnormal findings in such screenings were retinal hemorrhages [3]. As most retinal hemorrhages can absorb spontaneously, screening too early may increase patient’s economic burden due to multiple revisits. Further, it is very difficult to check for visual functions and eye movements like following light in neonates.

The purpose of this pilot study was to detect the prevalence of ocular abnormalities in infants around 6 weeks of age using red reflex examination and wide-field digital imaging system in a single public hospital and to explore the feasibility and efficacy of using wide-field digital imaging system as an universal tool for ocular screening in infants.

## Objectives and methods

Four hundred eighty-one infants who underwent the infant ocular screening program at Beijing Tongren Hospital affiliated to Capital Medical University between April 2015 and August 2016 were enrolled in the study. Infants who were unable to tolerate the eye screening examination conducted by the pediatrician or whose guardians refused to consent were excluded from the study. Clinical Research Ethics Committee of Beijing Tongren Hospital approved this study. All participants were screened according to the Declaration of Helsinki document on human research ethics, and underwent both verbal and written informed consent by their guardians for participation and data publication. The first screening time was around 6 weeks of age. The cost of one-time screening examination is about fifty US dollars. Two ophthalmologists who have been trained in pediatric retinal examinations performed the screening test and reviewed the images. A senior reviewer was consulted if the diagnoses were not consistent. Information about the infants’ gestational age at birth, birth weight, Apgar score and hypoxia during birth were collected. Details about the course of mother’s pregnancy and delivery were also obtained.

Vision assessment was performed initially by evaluating the ability to fix and follow light and objects binocularly and then monocularly. Following that an external eye examination, test for eye position, regular anterior segment examination and pupillary light reflex were performed. Red reflex examination was performed using a direct ophthalmolscope (mini3000, Heine, Germany). Pupils were then dilated by compound tropicamide eye drops (0.5% Tropicamide and 0.5% phenylephrine), which was administered every 10 min for 3 to 6 times until pupils dilated at least 5 mm. A wide-angle digital camera (RetCam 3, Clarity Medical System, CA, USA) and 130-degree lens were used to acquire anterior and posterior images from one eye to the other eye. Photographs of the fundus were taken in the following order: posterior pole, temporal, superior, nasal, and inferior retinal fields. Proparacaine hydrochloride eye drops were given for topical anesthesia before using the camera. Corneal lubricating gel and sterile pediatric eyelid speculum were used. Breast milk, formula, and clear liquids were withheld for 1 h before using the eyelid speculum. There were no ocular or systemic complications during or after any of the examination sessions.

Severity of retinal hemorrhage was graded by the number of hemorrhages per eye [4]. Presence of one or two hemorrhage spots was defined as grade 1, three to ten hemorrhage spots was defined as grade 2, and more than ten hemorrhage spots was defined as grade 3. Statistical analysis of the data was performed using a commercially available statistical software package (SPSS for Windows, version 17.0, SPSS Inc., Chicago, Illinois, USA). Data are presented as frequencies or as the mean ± SD. Logistic regression analysis was performed to identify the risk factors of abnormal findings. Probabilities of less than 0.05 were considered to indicate statistical significance.

## Results

Four hundred eighty-one infants were screened during the study period, including 253 (52.6%) males and 228 (47.4%) females. There were 408 (84.8%) full-term infants and 73 (15.2%) premature infants. Of all the infants screened, low birth weigh infants (birth weight less than 2500 g) were 50 (10.4%) and infants with macrosomia (birth weight equal or more than 4000 g) were 41 (8.5%). 276 (57.4%) were delivered vaginally and 205 (42.6%) were delivered by Caesarian section. 11 (2.3%) had hypoxia at birth (Apgar score less than 8) [5]. The birth weight of 73 preterm babies was 2400.0 ± 459.5 g, the gestational age was 35.2 ± 1.4 weeks, and the postmenstrual age was 41.5 ± 1.6 weeks. During the eighteen-month study period, 1785 newborns that met inclusion criteria for study participation were approached. Four hundred and eight-one subjects (962 eyes) participated in the universal ocular screening study, for a participation rate of 26.9%. The most common reasons why babies’ guardians chose not to participate in the study were they believed it was not necessary for the babies and they concerned about adverse effects.

**Fig. 1:**
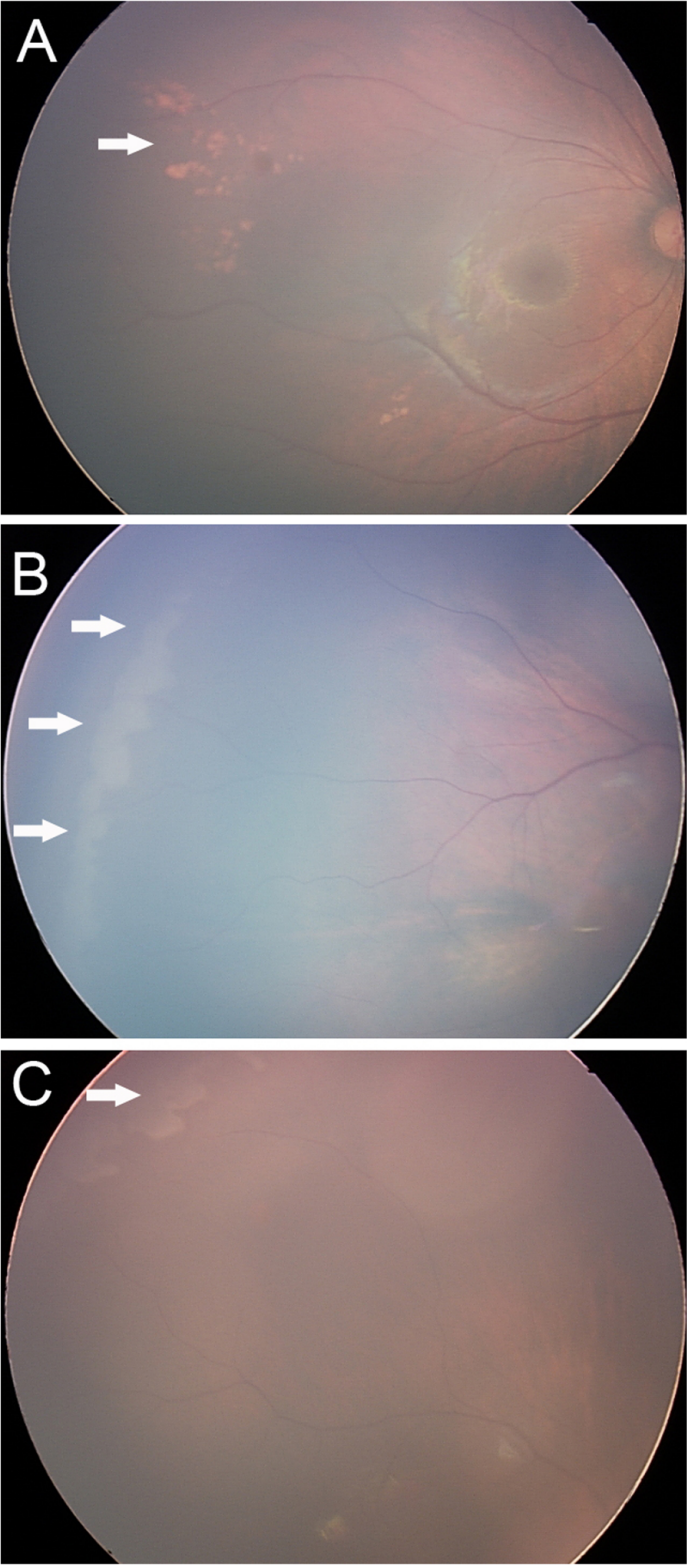
Retinal white changes were detected in at least one eye in 85 infants, accounting for 42.9% of abnormalities and 17.7% of all screened infants. **a**. Retinal white changes with spot shaped (pointing by white arrow). **b**. Retinal white changes with strip shaped (pointing by white arrows). **c**. Retinal white changes with patch shaped (pointing by white arrow)

**Fig. 2:**
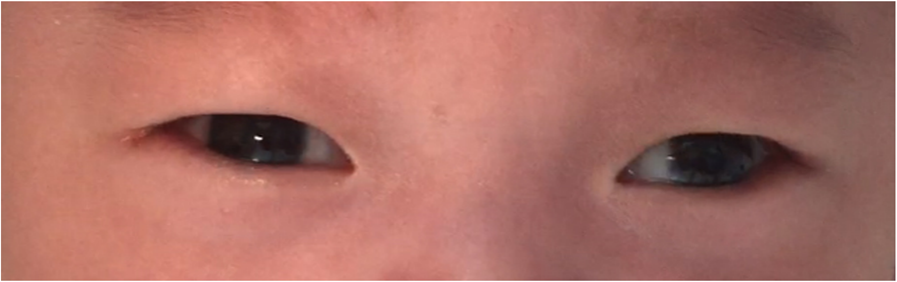
Concomitant exotropia was seen in 3.3% of infants screened

**Fig. 3:**
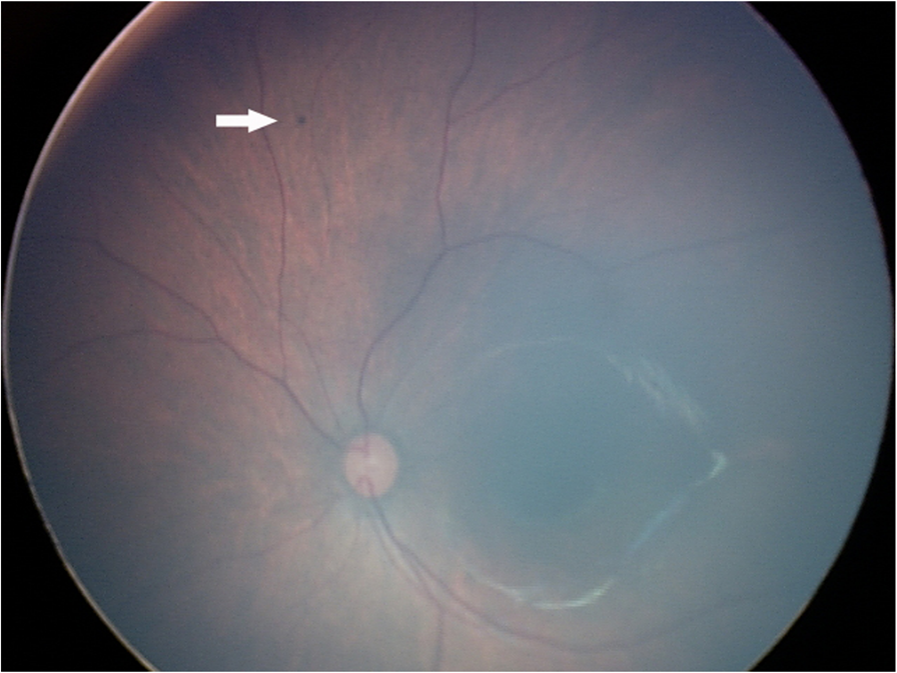
Retinal pigmentation was found in 3.3% of all screened infants (pointing by white arrow)

**Fig. 4:**
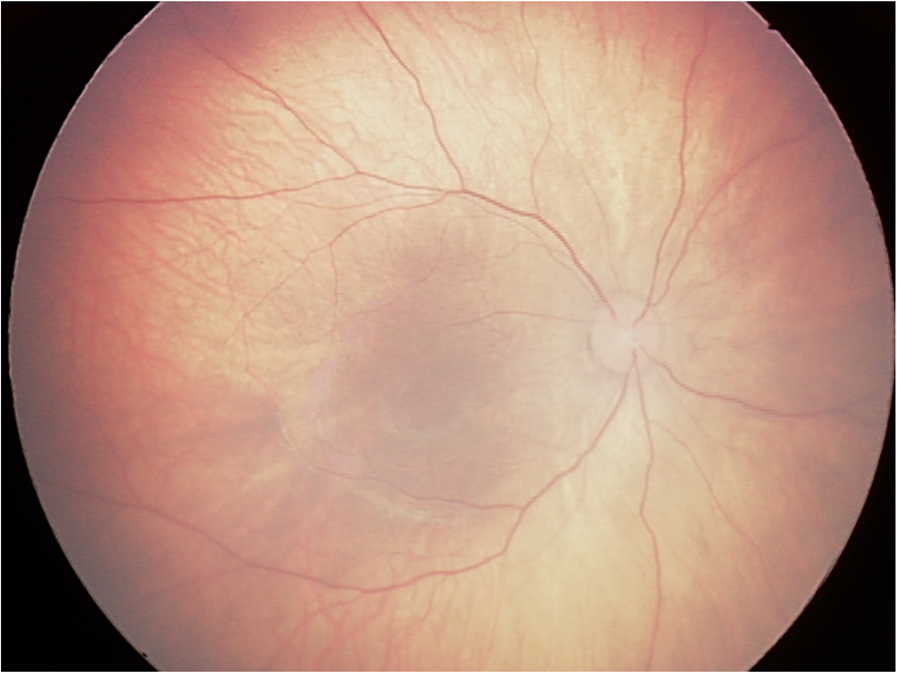
‘Albinism-like fundus’ was seen in 1.7% of all infants screened, who was excluded from albinism by Dermatologist consulting

**Fig. 5:**
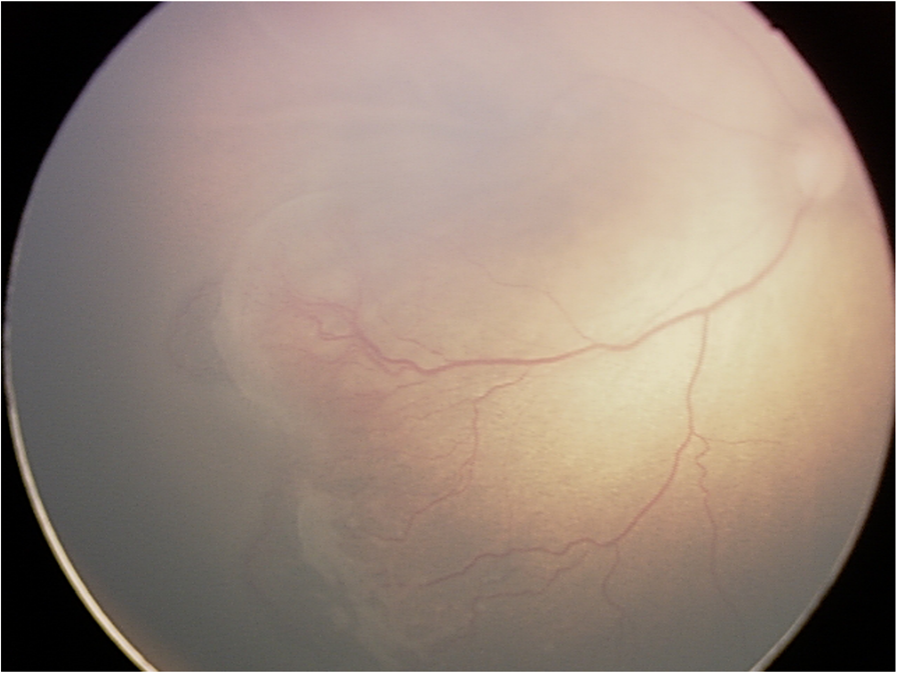
Threshold retinopathy of prematurity was detected and received laser treatment immediately

**Fig. 6:**
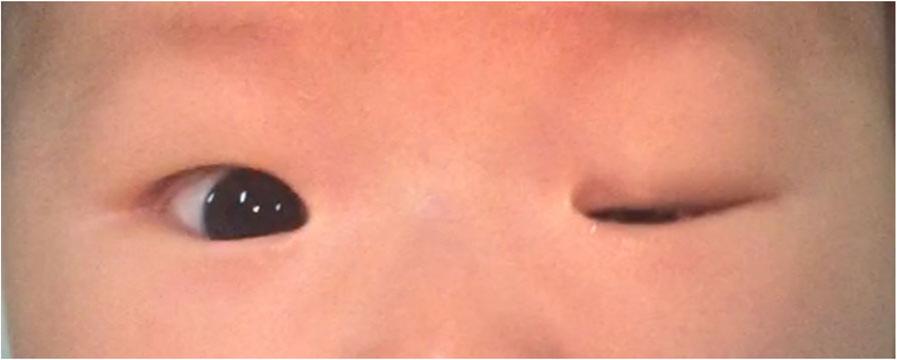
Ptosis was seen in one eye (0.2% of all screened infants)

Of the 481 infants who underwent eye examination, 198 (41.2%) were found to be abnormal. Retinal whites was the most common finding and was found in at least one eye in 85 infants, accounting for 42.9% of abnormalities and 17.7% of all screened infants (Fig. 1). Retinal hemorrhages were the second majority finding and were found in at least one eye in 32 infants, accounting for 16.2% of abnormalities and 6.7% of all screened infants. Other abnormalities were concomitant extropia (Fig. 2), retinal pigmentation (Fig. 3), neonatal dacryocystitis, ‘albinism-like fundus’ (Fig. 4), retinopathy of prematurity (ROP) (Fig. 5), congenital nasolacrimal duct obstruction, familial exudative vitreoretinopathy (FEVR), immature retina, corneal dermoid tumor, large physiologic cupping of optic disc, congenital persistent pupillary membrane, entropion trichiasis, subconjunctival hemorrhage, congenital cataract, vitreous hemorrhage, ptosis (Fig. 6) and choroidal nevus. These findings are summarized in Table 1. Intervention of any form was required in 22 infants, which accounted for 11.1% (22/198) of abnormalities detected and 4.6% (22/481) of all infants screened (Table 2). Once the ophthalmic diagnosis was made, 8 infants had to be referred to a dermatologist for specific examination towards any systemic condition that the infants could have possibly had.

**Table 1:** Ocular abnormalities detected in 198 cases out of 481 infants by eye screening

Abnormality	Abnormality cases (%)	Abnormality eyes (%)
Retinal white change	85(17.7%)	120 (12.5%)
Retinal hemorrhage	32 (6.7%)	39 (4.1%)
Concomitant exotropia	16 (3.3%)	32 (3.3%)
Retinal pigmentation	16 (3.3%)	19 (2.0%)
Neonatal dacryocystitis	13 (2.7%)	16 (1.7%)
Retinopathy of prematurity	9 (1.9%)	18 (1.9%)
Albinism-like fundus	8 (1.7%)	16 (1.7%)
Lacrimal duct obstruction	4 (0.8%)	6 (0.6%)
Familial exudative vitreoretinopathy	2 (0.4%)	4 (0.4%)
Immature retina	2 (0.4%)	4 (0.4%)
Corneal dermoid tumor	2 (0.4%)	2 (0.2%)
Large physiologic cupping of optic disc	2 (0.4%)	4 (0.4%)
Congenital persistent pupillary membrane	1 (0.2%)	2 (0.2%)
Entropion trichiasis	1 (0.2%)	2 (0.2%)
Subconjunctival hemorrhage	1 (0.2%)	1 (0.1%)
Congenital cataract	1 (0.2%)	1 (0.1%)
Vitreous hemorrhage	1 (0.2%)	1 (0.1%)
Ptosis	1 (0.2%)	1 (0.1%)
Choroidal nevus	1 (0.2%)	1 (0.1%)
Subtotal	198 (41.2%)	289 (30.0%)

**Table 2:** Characteristics of infants with diseases who were suggested interventions

Screening Number/Sex/Laterality	Diagnosis	Intervention Method
025/M/OS	Corneal dermoid tumor	Surgery
030/F/OS	Ptosis	Surgery
077/F/OD	CNLDO	Lacrimal sac massage
093/M/OD	Dacryocystitis	Lacrimal sac massage and topical antibiotics
146/F/OD	Dacryocystitis	Lacrimal sac massage and topical antibiotics
148/F/OS	Dacryocystitis	Lacrimal sac massage and topical antibiotics
176/F/OU	CNLDO	Lacrimal sac massage
252/M/OU	Dacryocystitis	Lacrimal sac massage and topical antibiotics
268/F/OD	Congenital Cataract	Surgery
275/F/OS	Dacryocystitis	Lacrimal sac massage and topical antibiotics
287/M/OS	Dacryocystitis	Lacrimal sac massage and topical antibiotics
294/F/OD	Dacryocystitis	Lacrimal sac massage and topical antibiotics
304/F/OU	ROP	Photocoagulation
342/F/OU	Dacryocystitis	Lacrimal sac massage and topical antibiotics
345/F/OS	Dacryocystitis	Lacrimal sac massage and topical antibiotics
392/F/OD	Dacryocystitis	Lacrimal sac massage and topical antibiotics
394/M/OU	Dacryocystitis	Lacrimal sac massage and topical antibiotics
403/F/OD	Dacryocystitis	Lacrimal sac massage and topical antibiotics
424/F/OU	CNLDO	Lacrimal sac massage
440/F/OS	Dacryocystitis	Lacrimal sac massage and topical antibiotics
445/F/OD	Corneal dermoid tumor	Surgery
474/F/OS	CNLDO	Lacrimal sac massage

Abbreviations: *M* male, *F* female, *OD* right eye, *OS* left eye, *OU* both eyes, *CNLDO* congenital nasolacrimal duct obstruction, *ROP* retinopathy of prematurity

Of all retinal white changes, 56 cases (69 eyes) were spot shaped, 17 cases (30 eyes) were strip shaped and 12 cases (21 eyes) were patch shaped changes. 22 cases (25 eyes) had changes on the posterior retina and 63 cases (95 eyes) on peripheral retina. No eminence, vascular branching, tortuous vessel, avascular peripheral retina or retinal ridge changes were found in these eyes. Retinal white changes did not relate to sex, family history of high myopia, preterm delivery, low birth weight, macrosomia, fetal distress, history of hypoxia, method of delivery, abnormal stage during labor, pregnancy-induced hypertension, or gestational diabetes (*P* > 0.05). These infants were examined during revisit at 3 months, 6 months and 12 months of age. Excluding 10 individuals who refused for follow up examination, retinal white changes disappeared spontaneously in 58 infants (68.2%) at 3 months of age, in 14 infants (16.5%) at 6 months of age, and in 2 infants (2.4%) at 12 months of age. Only 1 case (1.2%) had the retinal white spot without any change at 12 months of age.

Of 32 cases and 39 eyes with retinal hemorrhages, 29 eyes (74.4%) had grade 1, 9 eyes (23.1%) had grade 2, and one eye (2.6%) had grade 3 hemorrhage. Only one eye had a hemorrhage spot on the macula. All retinal hemorrhages were absorbed spontaneously at 3 months follow-up. Vitreous hemorrhage of one infant had been absorbed spontaneously at 3 months follow-up without any intervention. Two infants had been diagnosed as FEVR after checking their parents’ eyes. Both of them were grade 1 [6]. Retinal lesions were stable and without any neovascularization or intervention when followed up to 12 months. All concomitant exotropia were intermittent, and relieved spontaneously at 3 months follow-up. ‘Albinism-like fundus’ was noted in 8 infants without family history of albinism or absence of pigment in the skin, hair, lashes and iris. Albinism was excluded after consultation with a dermatologist. Of 73 preterm infants, ROP was detected in 9 infants (18 eyes), and immature retina was detected in 2 infants (4 eyes). All of them did not have ROP screening before we screened. The stage and zone of ROP in these infants and their gestational age, birth weight are demonstrated in Table 3. One patient with both eyes had threshold ROP and received photocoagulation treatment. Red reflex examination was normal except for one case of unilateral vitreous hemorrhage and one case with congenital cataract.

**Table 3:** Demographic data of patients with retinopathy of prematurity

Screen Number	Gender	Laterality	Stage/Zone	Gestational age (weeks)	Birth Weight (grams)
053	Female	Both Eyes	2/III	32 ^+ 6^	1780
149	Male	Both Eyes	2/II	32 ^+ 5^	1640
161	Male	Both Eyes	1/III	34 ^+ 2^	1720
304	Female	Both Eyes	3+/I	31 ^+ 6^	1320
305	Female	Both Eyes	2/II	31 ^+ 6^	1420
311	Female	Both Eyes	1/III	35	2460
312	Female	Both Eyes	1/II	33 ^+ 5^	2130
342	Female	Both Eyes	1/III	36 ^+ 1^	2020
465	Male	Both Eyes	2/III	33 ^+ 4^	2280

The patient with vitreous hemorrhage of his right eye could not fix or follow a light or object at the first time screening. At his 3 months follow-up, his right eye with vitreous hemorrhage was absorbed spontaneously. At that time, he can fix and follow a light and object with left eye. But when we tried to assess the visual function of his right eye, he refused to cover his left eye and cannot corporate with fixing or following test. We believed that his right eye had visual impairment because of visual deprivation and suggested him to cover his left eye for two hours every day and asked him to re-check his visual function one month later, but the patient never came back and lost to follow-up.

## Discussion

Universal ocular screening of infants is not a common practice in most developing countries and even in some developed countries. Recently, national health and family planning commission of China has issued some guidelines about eye screening at an early age. But only a few children can be screened in some big cities. Those who lived in less developed areas cannot be covered by the healthcare system. Red reflex examination is recommended in neonates, infants and children in several developed countries [7, 8]. But red reflex examination still has some limitations in detecting sensitivity of fundus abnormality [9]. In this study, we also found 157 abnormal cases by RetCam but normal by red reflex examination.

The ocular abnormalities can be divided into three types as following: The first type is with no particular clinical significance, such as subconjunctival hemorrhage, retinal pigmentation and self-resolved retinal hemorrhage. The second type is with some clinical significance that should be monitored periodically through revisit check-ups, which include immature retina, FEVR grade 1 and grade 2 and retinal white change. The third part is with important clinical significance and requires some form of intervention, like threshold ROP, dacryocystitis, severe cataract and ptosis. In this pilot study, we found 198/481 (41.2%) had abnormal ocular signs, and 22/481 (4.6%) required at least one form of intervention. Those who required intervention were healthy and normal at the time of universal eye screening.

Congenital nasolacrimal duct obstruction is the most common disorder of the nasolacrimal duct system in infants. Although the diagnosis of a congenital nasolacrimal duct obstruction is usually not difficult, there are still some debatable issues about the timing and methods of intervention among pediatric ophthalmologists. In this study, we prescribed lacrimal sac massage or topical antibiotics for infants with congenital nasolacrimal duct obstruction and dacryocystitis as nonsurgical interventions, which are the same as Pediatric Eye Disease Investigator Group did [10]. Although most nasolacrimal duct obstruction can be self-resolved during the first year of life, and there is no proof that massage can elevate the recovery rate, it still seems logical to perform massage as a noninvasive intervention. A survey of common management policies for nasolacrimal duct obstruction among pediatric ophthalmologists showed that 82% respondents preferred to instruct parents to massage nasolacrimal duct during the first year of life [11]. In addition, more than 90% of pediatric ophthalmologists in that survey wait until approximately 1 year old before recommending a surgical intervention for congenital nasolacrimal duct obstruction.

The previous studies of universal eye screening were based on neonates within 72 h after birth [2, 12, 13]. The most common ocular abnormality in their studies was retinal hemorrhage with 21.52%, 2.4%, and 20.3% prevalence respectively. In this pilot study, the prevalence of retinal hemorrhage is 6.7%, and all cases spontaneously resolved by the 3rd month revisit check-ups. The prevalence of subconjunctival hemorrhage is 0.2% compared to 1.4% in previous study [2]. The lower prevalence of retinal hemorrhage and subconjunctival hemorrhage in our study is due to an older age at the time of examination compared to the other study. We believe that ocular examination at an older age can reduce revisit times of the self-resolving hemorrhages. Furthermore, fixation and following light and object can be assessed in infants who are 6 weeks old. In this study, we found only one eye with vitreous hemorrhage that could not fix or follow a light or object.

Congenital cataract is an important treatable disease leading to childhood visual disability. Universal ocular screening is an effective to screening congenital cataracts at an early age. To avoid amblyopia and nystagmus caused by visual deprivation, early intervention is recommended [14]. In infants suffering from congenital cataracts, especially posterior, perinuclear, nucleus, and total cataracts, the crucial time for surgery in order to preventing amblyopia is probably within the first three months of life [15]. FEVR, characterized by congenital anomalous retinal vascularization, is an inherited vitreoretinopathy. The presentation and severity of this disease could be very different even in the same family. The mild and most common lesions often manifest as avascular zone in peripheral retina, supernumerous vascular branching, venous-venous anastomoses and vitreoretinal adhesions, which could be asymptomatic during the entire life. The severe and progressive forms of the diseases are including neovascularization, retinal exudates, retinal hemorrhage, preretinal membranes, retinal folds and retinal detachment [6, 16]. In this study, we found two infants with bilateral avascular peripheral retinas and finally were diagnosed as FEVR. Fluorescein angiogram (FA) is the most helpful method to confirm and diagnose FEVR, but during routine eye screening we cannot perform FA on the 6 week-old infants under topical anesthesia, so we made the diagnosis based on demographic features, clinical presentations, fundus findings and family history through the following steps. First, we differentiated them from ROP by their full term birth and normal birth weight without supplemental oxygenation. Second, incontinentia pigmenti and Norrie disease can be excluded by their skin manifestations, hearing screening results, genders and family histories. Third, the fundus showed classic vascular anomalies such as avascular zone, supernumerous vascular branching and peripheral exudation, which can be differentiated from avascular retina. Forth, when these two patients were suspicious for FEVR, we checked their parents’ fundus by ophthalmoscopy and FA. Two mothers were diagnosed as FEVR, and one of them had bilateral retinal holes and received photocoagulation treatment. Clinical examination alone can be insufficient to identify the subtle and atypical vascular changes of FEVR, which requiring the assistance of FA. Fortunately, the two FEVR patients had classic vascular changes, in addition to the demographic features and positive familial presentations that provided further evidence for the diagnosis. FEVR can be found through universal ocular screening and treated timely to avoid visual impairment consequences. Considering that FEVR is a lifelong disease, FEVR patients should be followed up regularly.

Retinal white spots or area was the most common finding, which was not mentioned in previous studies. All retinal white changes in the posterior area were dot shaped. Peripheral retinal white changes can be spots, strips or patches, and some of them were adjacent to ora serrata. We hypothesize that several reasons could cause this kind of retinal change. The first is because the peripheral retina has poor blood flow as retinal vessels are developing, which causes uneven cellular metabolism and displays retinal malnutrition-like changes. The second speculation is that the development of retinal vessel epithelial cells at the periphery is delayed than the posterior areas, which cause retinal exudations due to the immature development of blood-retina barrier. In addition, subretinal lipid or calcium deposition partially absorbed retinal hemorrhage and local undeveloped retinal pigment epithelium may also attribute to these findings. Undoubtedly, FA is the only way to ascertain the nature of these white changes. But we cannot perform FA on the 6 week-old infants under topical anesthesia during routine eye screening. Therefore, we use rule-out and observation strategy for these findings. Firstly, we ruled-out the potential diseased which could cause visual impairment like ROP, FEVR and retinoblastoma by checking if the white changes were in the vascular area, if the peripheral vascular branching and position are normal, if the white change was protruded into vitreous cavity. Secondly, for the retinal white changes without potential risks, we can wait and observe to find out what they would be. Most retinal white changes were relieved spontaneously as the children grew, and were not related to mother or newborn conditions, so it can be considered as a physiological change. In this study, only one case of a retinal white spot at posterior area remained unchanged at 12 months revisit. This spot was with a clear boundary and without eminence. We considered it as retinal pigment maldistribution. Above were our hypotheses because only retinal FA can ascertain the nature of these changes, which need further researches.

Choroidal vessels can be seen in ‘albinism-like fundus’. But none of them had family history of albinism or absence of pigment in the skin, hair, eyelashes and iris. We considered immature retinal pigment epithelial cells to be the etiological factor for ‘albinism-like fundus’. All concomitant exotropia were relieved spontaneously by the revisit at 3 months of age, which reflects immaturity of the ocular motor system as the possible etiology [17].

There are certain limitations in this study. Firstly, it is a single center study in a public hospital and it is a pilot study as the sample size is not large. As there is no control group in this study, it is more important to discuss ‘how to explain what we find’ than report ‘what we find’. Secondly, further in-depth study involving multiple centers with large sample size and long time follow-up needs to be done in order to discuss the prevalence and etiology of ocular abnormalities.

## Conclusion

To sum up, universal ocular screening is not only necessary for preterm infants but also for full-term infants. In addition to red reflex examination, wide-field digital imaging can enhance the sensitivity of screening ocular fundus abnormities. First screening at around six weeks of age could reduce re-examinations for self-resolved cases. Visual function and eye position can also be checked at this age. The outcomes of our work would help in revising guidelines for children’s health screening system.
